# Intestinal Epithelial Cell-Derived Extracellular Vesicles Modulate Hepatic Injury via the Gut-Liver Axis During Acute Alcohol Injury

**DOI:** 10.3389/fphar.2020.603771

**Published:** 2020-12-21

**Authors:** Arantza Lamas-Paz, Laura Morán, Jin Peng, Beatriz Salinas, Nuria López-Alcántara, Svenja Sydor, Ramiro Vilchez-Vargas, Iris Asensio, Fengjie Hao, Kang Zheng, Beatriz Martín-Adrados, Laura Moreno, Angel Cogolludo, Manuel Gómez del Moral, Lars Bechmann, Eduardo Martínez-Naves, Javier Vaquero, Rafael Bañares, Yulia A. Nevzorova, Francisco Javier Cubero

**Affiliations:** ^1^ Department of Immunology, Ophthalmology and ENT, Complutense University School of Medicine, Madrid, Spain; ^2^ 12 de Octubre Health Research Institute (imas12), Madrid, Spain; ^3^ Servicio de Aparato Digestivo del Hospital General Universitario Gregorio Marañón, Instituto de Investigación Sanitaria Gregorio Marañón (IiSGM), Madrid, Spain; ^4^ Department of Hepatobiliary Surgery, Nanjing Drum Tower Hospital, The Affiliated Hospital of Nanjing University Medical School, Nanjing, China; ^5^ Centro Nacional de Investigaciones Cardiovasculares Carlos III, Madrid, Spain; ^6^ Bioengineering and Aerospace Engineering Department, Universidad Carlos III de Madrid, Madrid, Spain; ^7^ Centro de Investigación Biomédico en Red de Salud Mental (CIBERSAM), Madrid, Spain; ^8^ Department of Internal Medicine, University Hospital Knappschaftskrankenhaus, Ruhr-University Bochum, Bochum, Germany; ^9^ Department of Gastroenterology, Hepatology, and Infectious Diseases, Otto von Guericke University Hospital Magdeburg, Magdeburg, Germany; ^10^ Centre for Biomedical Research, Network on Liver and Digestive Diseases (CIBEREHD), Madrid, Spain; ^11^ Department of General Surgery, Hepatobiliary Surgery, Ruijin Hospital, Shanghai Jiao Tong University School of Medicine, Shanghai, China; ^12^ Department of Anesthesiology, Zhongda Hospital, School of Medicine, Southeast University, Nanjing, China; ^13^ Department of Pharmacology and Toxicology, Complutense University School of Medicine and Centre for Biomedical Research, Network on Respiratory Diseases (CIBERES), Madrid, Spain; ^14^ Department of Cell Biology, Complutense University School of Medicine, Madrid, Spain; ^15^ Department of Internal Medicine III, University Hospital RWTH Aachen, Aachen, Germany

**Keywords:** hepatocytes, intestinal epithelial cells, extracellular vesicles, alcohol (EtOH), gut-liver axis

## Abstract

Binge drinking, i.e., heavy episodic drinking in a short time, has recently become an alarming societal problem with negative health impact. However, the harmful effects of acute alcohol injury in the gut-liver axis remain elusive. Hence, we focused on the physiological and pathological changes and the underlying mechanisms of experimental binge drinking in the context of the gut-liver axis. Eight-week-old mice with a C57BL/6 background received a single dose (p.o.) of ethanol (EtOH) [6 g/kg b.w.] as a preclinical model of acute alcohol injury. Controls received a single dose of PBS. Mice were sacrificed 8 h later. In parallel, HepaRGs and Caco-2 cells, human cell lines of differentiated hepatocytes and intestinal epithelial cells intestinal epithelial cells (IECs), respectively, were challenged in the presence or absence of EtOH [0–100 mM]. Extracellular vesicles (EVs) isolated by ultracentrifugation from culture media of IECs were added to hepatocyte cell cultures. Increased intestinal permeability, loss of zonula occludens-1 (ZO-1) and MUCIN-2 expression, and alterations in microbiota—increased *Lactobacillus* and decreased Lachnospiraceae species—were found in the large intestine of mice exposed to EtOH. Increased TUNEL-positive cells, infiltration of CD11b-positive immune cells, pro-inflammatory cytokines (e.g., *tlr4*, *tnf*, *il1β*), and markers of lipid accumulation (Oil Red O, *srbep1*) were evident in livers of mice exposed to EtOH, particularly in females. *In vitro* experiments indicated that EVs released by IECs in response to ethanol exerted a deleterious effect on hepatocyte viability and lipid accumulation. Overall, our data identified a novel mechanism responsible for driving hepatic injury in the gut-liver axis, opening novel avenues for therapy.

## Introduction

Alcohol abuse is a leading cause of liver-related morbidity and mortality, which has become a global problem due to the financial burden on society and the healthcare system (Dolganiuc and Szabo, 2009; Gao and Bataller, 2011). While the adverse effects of long-term chronic alcohol abuse have been widely studied, the underlying mechanisms of short-term binge and sporadic drinking, also termed acute alcohol-derived tissue injury, remain elusive.

Binge drinking refers to an excessive consumption of large amounts of alcohol in a very short period of time, in which increases the blood alcohol concentration (BAC) levels to at least 0.08 g/dl (Ventura-Cots et al., 2017). The National Institute on Alcohol Abuse and Alcoholism defines binge drinking as a consume of four (women) or five (men) standard drinks per day within 2 h at least once during Binge drinking refers to excessive consumption of large amounts of alcohol in a short period of time, in which the (BAC) levels increase to at least 0.08 g/dl (Ventura-Cots et al., 2017). The National Institute on Alcohol Abuse and Alcoholism defines binge drinking as the consumption of four (women) or five (men) standard drinks per day within 2 h at least once during the past 30 days (Ventura-Cots et al., 2017). However, the standard drink varies significantly from country to country from 7.9 g of alcohol in the United Kingdom to 14 g in the United States or 19.75 g in Japan (Dolganiuc and Szabo, 2009).

Noticeably, the rate of alcohol absorption depends on several factors, including the amount and concentration of alcohol ingested and physiological factors determined by gender (Cederbaum, 2012; Kim et al., 2015; Griswold et al., 2018). Furthermore, sex differences such as body fat, body water, levels of alcohol dehydrogenase (ADH), and hormones affect the hepatic metabolism of alcohol (Gill, 2000; Naugler et al., 2007; Griswold et al., 2018; Lamas-Paz et al., 2018).

Upon acute alcohol injury, alterations of the intestinal epithelial barrier occur at multiple levels including tight junctions (TJs), between gut epithelial cells, production of mucin, recruitment and activation of inflammatory cells to the intestinal wall. In addition, the composition of the gut microbiome changes as a result of alcohol consumption. These result in increased translocation of microbial product from the gut to the liver via the portal circulation. Besides increased levels of lipopolysaccharides, other microbial components may also reach the liver where in the liver sinusoids Kupffer cells and other recruited immune cells become activated and produce large amounts of pro-inflammatory cytokines (e.g., TNF, IL-1β), which further increase gut permeability, thus fueling inflammation in the gut and favoring the development of liver disease (Szabo, 2015; Gao et al., 2019).

Emerging evidence showed that EVs are important contributors to the coordinated signaling events between the gut and the liver (Bui et al., 2018), a bidirectional axis linking the biliary tract, the portal vein, and the systemic circulation (Tripathi et al., 2018).

In the present study, we aimed to evaluate the underlying mechanisms driving acute alcohol injury *in vitro* and in female and male mice in the context of the gut-liver axis.

## Materials and Methods

### Cell Culture and Cell Viability

HepaRG cells (BioPredict International, Rennes, France) were seeded following the supplier’s protocol, and Caco-2 cells, a human intestinal cell line widely used as a model of the intestinal barrier, were cultured in Dulbecco’s modified Eagle’s medium (Gibco, Rockville, MD) containing 20% heat-inactivated FBS, 1% penicillin/streptomycin, and 2 mM l-glutamine (ICN Pharmaceuticals, Costa Mesa, CA). Once HepaRGs or Caco-2 cells reached approximately 85% confluence, starving was performed for 4 h, and cells were challenged with EtOH [0–100 mM] (Panreac AppliChem, Darmstadt, Germany) for another 24 h. Cells were kept at 37°C in an atmosphere with 5% CO_2_. Pictures of cells were taken in an optical microscope (DMIL LED, Leica, Wetzlar, Germany) connected to a camera (Leica MC170HD, Leica). Cell viability was determined by CCK8 (Merck, Munich, Germany) following the manufacturer’s instructions. After fixing the cells with 4% PFA, cell death and lipid accumulation were tested by TUNEL (Roche, Rotkreuz, Switzerland) and oil red O (ORO) (Merck), respectively, as previously described (Cubero et al., 2015; Liao et al., 2019).

### Animal Model of Acute Alcohol Injury

Healthy C57BL/6J mice purchased from ENVIGO (Valencia, Spain) were bred and maintained in the Animal Facility of the Faculty of Biology at UCM, Madrid, in a temperature-controlled room with 12 h light/dark cycles and allowed food and water *ad libitum*. For our study, we used female and male 8 week-old mice. Animal studies were approved by the local authority (Consejería de Medio Ambiente, Administración Local y Ordenación del Territorio; PROEX-154/16).

Acute alcohol injury was performed by oral gavage. Briefly, mice (*n* = 8–10/group) were fasted overnight for 12 h. In the morning, they were fed with a dose of 30% EtOH (gavage of 6 g/kg b.w.) (Pruett et al., 2020) using a gavage needle (Kent Scientific, Torrington, CT). Mice fed with PBS instead of EtOH served as controls. All animals were sacrificed at 8 h after the EtOH challenge using an overdose of isoflurane (Solvet, Segovia, Spain) inhalation.

### Intestinal Permeability *In Vivo*

Isothiocyanate conjugated dextran (FITC-dextran, molecular mass 4.0 kDa) (TdBCons, Uppsala, Sweden) was dissolved in PBS at a concentration of 200 mg/ml and administrated to 12 h fasted mice (10 ml/kg body weight) using a gavage needle (Kent Scientific). After 4 h, mice were sacrificed by an overdose of isoflurane (Solvet) inhalation. Concentration of FITC was determined in serum by fluorometry with an excitation of 485 nm and an emission wavelength of 528 nm using serially diluted FITC-dextran (0, 125, 250, 500, 1,000, 2,000, 4,000, 6,000, 8,000, 10,000 ng/ml) as standards.

### Histological and Morphological Analyses

Livers from mice were harvested, fixed with 4% PFA, and embedded in paraffin for histological evaluation using hematoxylin and eosin (H&E), performed by Dr Juana Flores, an experienced pathologist (School of Veterinary, UCM). Photomicrographs of stained sections were randomly taken in a ×20 magnification in an optical microscope (Nikon Eclipse Ci, Tokyo, Japan), and ORO-positive areas were quantified using free NIH Image/J software (National Institutes of Health, Bethesda, MD).

### Immunofluorescence Staining

Liver and colon from each mouse were preserved in cassettes in Tissue-Tek (Sakura Finetek U.S.A, Torrance, CA) at −80 C. Immunostainings for ZO-1 (Invitrogen, Paisley, FL), MUCIN-2 (Santa Cruz, Dallas, TX), TUNEL (Roche), and CD11b (BD, Madrid, Spain) were performed as previously described (Liao et al., 2019). Anti-mouse and anti-rabbit Alexa Fluor 488 (Invitrogen) were used as secondary antibodies.

### Quantitative Real-Time Polymerase Chain Reaction

Total RNA was purified from liver tissue using Trizol reagent (Invitrogen). Total RNA [1 µg] was used to synthesize cDNA using Super Script first Stand Synthesis System (Invitrogen) and was resuspended in 100 µl of RNAse-free water (Merck). Quantitative real-time PCR was performed using SYBR Green Reagent (Invitrogen) by the Genomics and Proteomics Facility (School of Biology, UCM). The mRNA expression of *il1β*, *Srebp1*, *Tlr4*, *Tnf*, and *Gapdh* expression was studied (Supplementary Table S1). Relative gene expression was normalized to the expression of *Gapdh*. Primer sequences are provided upon request.

### Microbiota Analysis

DNA was extracted from foecal content as previously described (Sydor et al., 2020). All samples were resampled to the minimum sequencing depth of 17719 reads using phyloseq package (Mcmurdie and Holmes, 2013) and returning 2,167 phylotypes (Supplementary Figure S2).

### Biochemical Measurements

Serum transaminases in blood and cell’s supernatant were analyzed in the Central Laboratory Facility at the Gregorio Marañón Research Health Institute at Madrid (iISGM) using automated analyzers. For the evaluation of intrahepatic triglycerides, liver samples were homogenized in a specific Tris buffer (10 mM Tris, 2 mM EDTA, 0.25 M sucrose, and pH 7.5) and successively processed using a commercial colorimetric kit (Human Diagnostics, Wiesbaden), according to the manufacturer’s instructions.

### Isolation of Extracellular Vesicles

For *in vivo* experiments, blood from the cava vein was collected in 1.1 ml serum gel polypropylene microtubes (Sarstedt, Barcelona). Serum was obtained by centrifugation at 12,000 *g* for 10 min at 4°C. Serum was then centrifuged using a Microliter Centrifuge Z233 MK-2 (Hermle, Wehingen, Germany) at 10,000 *g* during 30 min. Then, the supernatant was ultracentrifuged twice at 100,000 *g* using a Hitachi micro ultracentrifuge CS150FNX (Hitachi, Tokyo, Japan) with an S5AA2 rotor in 1.5 ml Eppendorf tubes (Fisher Scientific, Madrid, Spain) during 75 min each. Finally, the pellet containing EVs was resuspended in 50 µl of PBS. Samples were measured by dynamic light scattering (Malvern Instruments, United Kingdom). Next, recollected samples were filtered through 0.44 µm filters, and the obtained sample was diluted 1:10 for nanoparticle tracking analysis (NTA).

For *in vitro* tests, supernatants were collected from cultures of Caco-2 and HepaRG cells treated with EtOH [0–100 mM]. EVs were isolated from supernatants using ultracentrifugation. Briefly, the culture supernatant was centrifuged at 2,000 *g* and 10,000 *g* for 20 and 30 min, respectively, to remove cellular debris and larger vesicles. The resultant supernatant was ultracentrifuged twice at 100,000 *g* for 75 min. The pellet was resuspended in 50 µl of PBS and stored at −80°C. Free EVs medium was prepared by ultracentrifugation of 10% FBS for 16 h on a Hitachi micro ultracentrifuge CS150FNX (Hitachi, Japan).

### Experiments With Extracellular Vesicles

HepaRG cells were cultured with EVs isolated from the supernatant of Caco-2 cells previously treated with EtOH [0–100 mM] and diluted into EVs-free medium. Controls were challenged with EVs-free medium. Twenty-four hours later, cells were washed and fixed with 4% PFA. Cell viability, cell death, and lipid deposition were then evaluated.

### Nanoparticle Tracking Analysis

The number and size of EVs were quantified and characterized using NTA with the Nanosight NS300 instrument equipped with a 405 nm green laser and an sCMOS high sensitivity camera with NTA (Malvern Instruments). Particles were recorded in five different 60-s videos. The data were analyzed using NTA software SOPs v3.4, following the manufacturer’s instructions.

### Statistical Analysis

All statistical analyses consisted of One-Way ANOVA followed by Tukey post-hoc test using GraphPad Prism version 8.0 software (San Diego, CA). A *p* < 0.05 was considered statistically significant. Data were expressed as mean ± SD of the mean (SEM).

## Result

### Experimental Acute Alcohol Injury Induces Gut Dysbiosis

**FIGURE 1:**
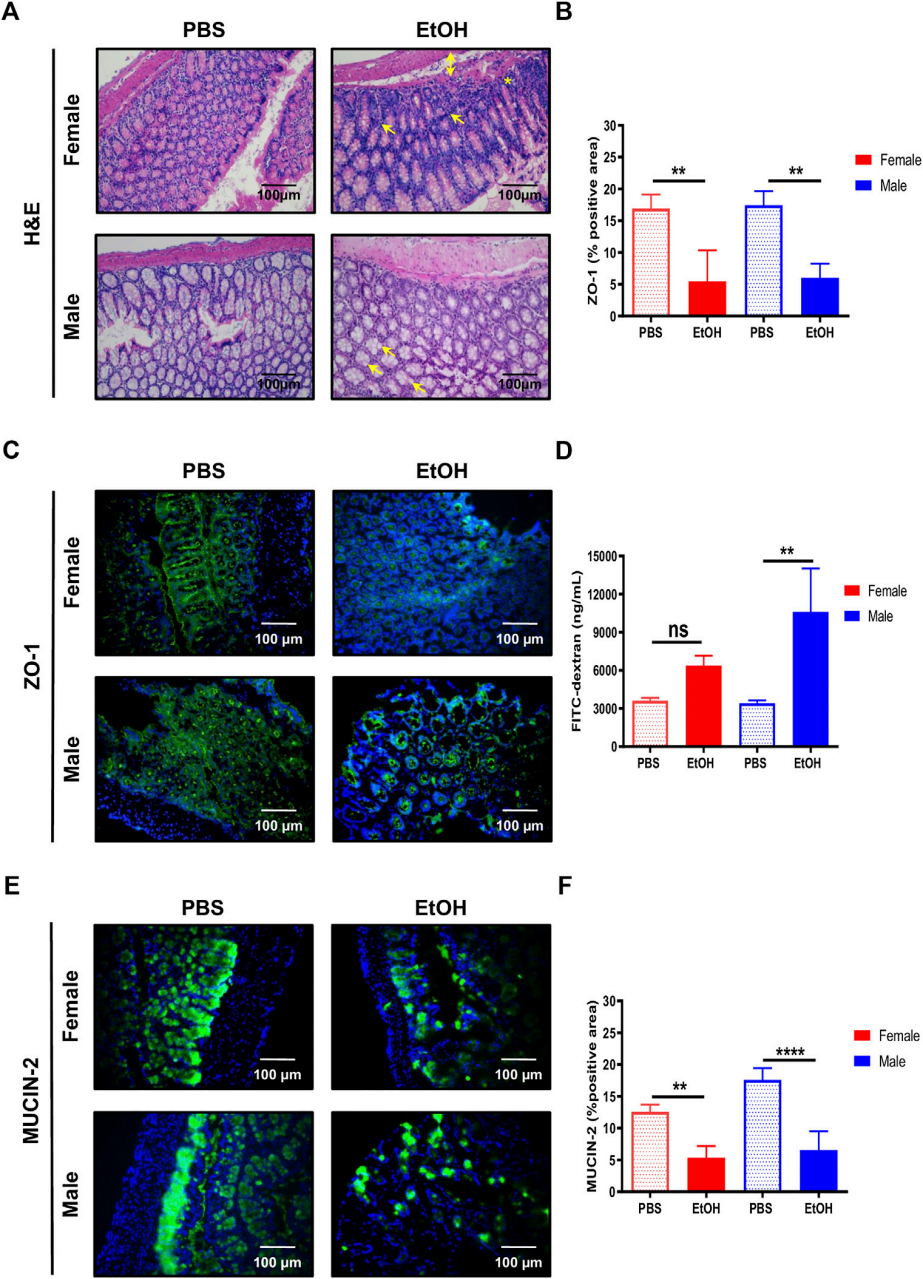
Acute alcohol injury disrupts the gut intestinal barrier in female and male mice. **(A)** Representative H&E staining was performed in paraffin colon sections of female and male mice treated with EtOH [6 g/kg] or PBS (*n* = 5–7/group). **(B)** Quantification of the ZO-1 positive area was performed and graphed (*n* = 4/group). **(C)** ZO-1 immunofluorescence staining was performed in colons of male and female mice. **(D)** FITC-dextran levels in serum from female and male mice was quantified as a measure of intestinal permeability (*n* = 3–4/group). **(E)** MUCIN-2 immunofluorescence staining was performed in colons of female and male mice. **(F)** Quantification of the MUCIN-2 positive area was done and graphed (*n* = 4–5/group; **p < 0.01, ****p < 0.0001). In female mice, arrows mark mixed inflammation; double arrow, oedema in submucosa and a star, degeneration of crypt basal cells. In male mice, arrows mark mild hypertrophy in Goblet cells.

The gut mucosa is particularly susceptible to alcohol-induced tissue injury (Molina and Nelson, 2018). Thus, we first focused on evaluating the impact of binge alcohol drinking via the circulatory system in the large intestine. Histopathological evaluation of experimental acute alcohol injury revealed mixed inflammation in the colon, oedema in the submucosa, and cellular degeneration of crypt basal cells in colons of female mice exposed to acute alcohol injury (Figure 1A). In contrast, male-EtOH treated colon displayed mild hypertrophy in Goblet cells (Figure 1A). No relevant findings were observed in mice challenged with PBS (Figure 1A).

Since acute alcohol exposure induces gut permeability by disrupting not only the epithelial cells, but also the space between them that is controlled by (TJs) (Fanning and Anderson, 2009), we next evaluated whether changes in intestinal TJs that result in leaky gut occurred during experimental acute alcohol injury in female and male mice. Interestingly, the expression of zonula occludens-1 (ZO-1) was significantly decreased in colons of female and, to a bigger extent, of male mice, compared with PBS-fed animals (Figures 1B,C).

Next, we used the FITC-dextran gavage method to determine the impact of acute alcohol injury on intestinal permeability *in vivo*. Compared with PBS-treated animals, intestinal permeability tended to increase in female mice and was significantly increased in male mice exposed to EtOH (Figure 1D). Altogether, these results indicated that experimental acute alcohol injury increased intestinal permeability in mice.

The intestinal mucous layer prevents direct contact between the intestinal epithelium and bacteria by avoiding its way through. The major component of the intestinal mucous layer is MUCIN-2, which is secreted by Goblet cells (Van Der Sluis et al., 2006). Immunofluorescent staining of MUCIN-2 revealed thinner layers in the large intestine of EtOH-treated female and, to a bigger extent, male mice, compared with PBS-treated animals (Figures 1E,F).

### Characterization of Changes in Gut Microbiota During Acute Alcohol Injury

Overwhelming evidence has shown that changes in the intestinal microbiome might contribute to alcohol-associated intestinal inflammation and permeability (Sarin et al., 2019). Therefore, foecal microbiota from all experimental groups was analyzed (Supplementary Figures S1A,B). No difference in gut microbiota composition was found between female or male mice treated with either PBS or EtOH (*p* value = 0.049). Principal coordinates analysis (Supplementary Figure S1A) revealed that six phylotypes, four of them belonging to the phylum Firmicutes, trended to increase under acute ethanol consumption. On the contrary, 12 phylotypes, seven belonging also to Firmicutes, trended to diminished in EtOH-treated mice. Especially Phy1 and Phy5 (both belonging to the family Erysipelotrichaceae) together with Phy2 (*Lactobacillus* sp.) were observed in more abundance in EtOH-treated mice, while Phy3 (*Turicibacter* sp*.*), Phy7 (*Bifidobacterium pseudolongum*), and Phy9 (*Lactobacillus johnosonii* or *acidophilus*) were observed in less abundances in EtOH-treated mice (Supplementary Figure S1B). Mann-Whitney test detected statistically significant differences between EtOH-treated and PBS-treated mice in three phylotypes. Phy2 (*p* value = 0.006) and Phy5 (*p* value = 0.006), both belonging to *Lactobacillus* sp., increased the relative abundance in EtOH-treated mice, while Phy63 (*p* value = 0.03), belonging to Lachnospiraceae, diminished in EtOH-treated mice.

Altogether, these results suggest that experimental acute alcohol-derived tissue injury triggers gut dysbiosis.

### Steatosis and Inflammation Induced by Acute Alcohol Injury Is More Pronounced in Female Mice

**FIGURE 2:**
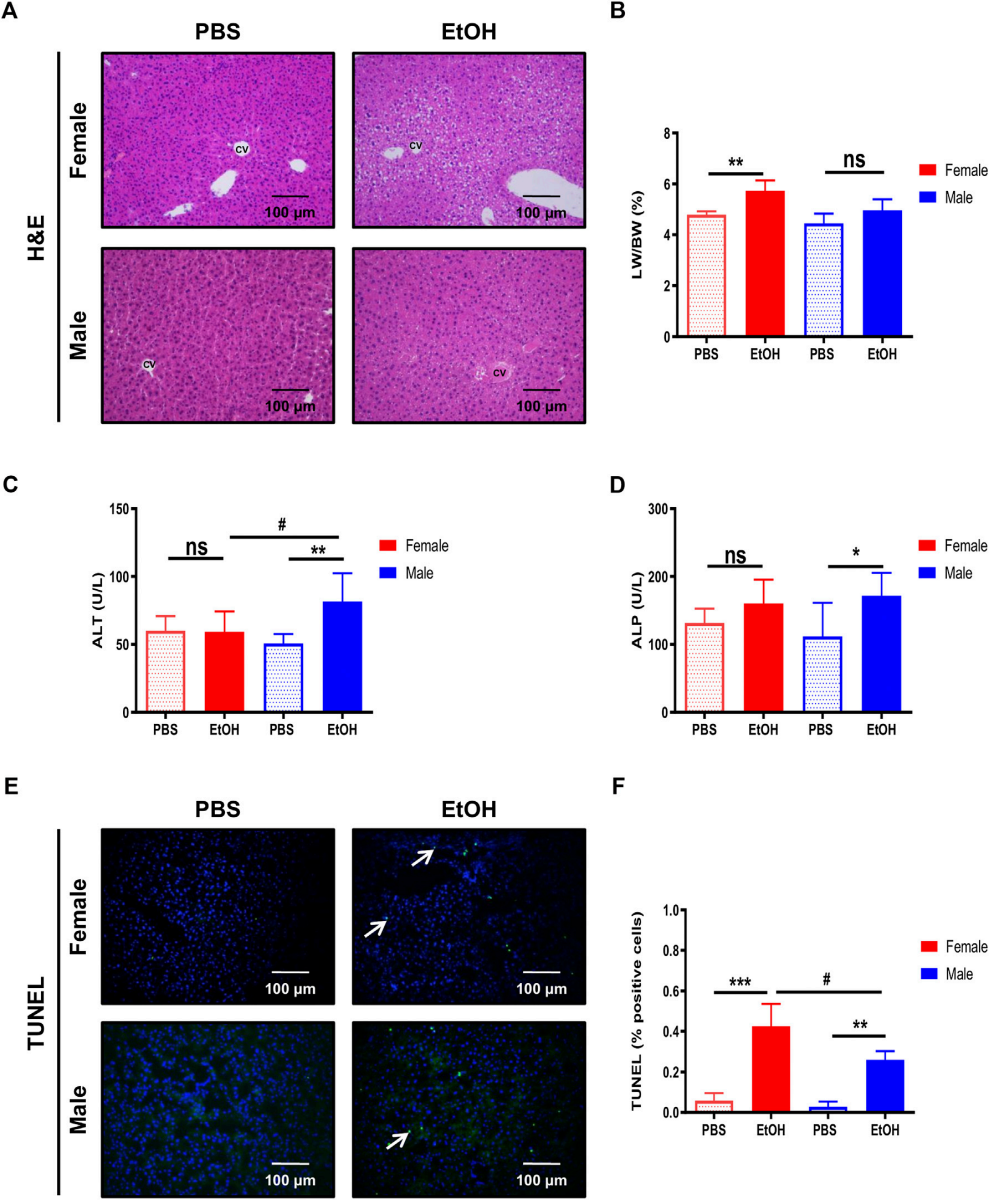
Binge ethanol exposure to mice causes changes in liver architecture and cell death. **(A)** H&E staining in female and male mice treated with EtOH [6 g/kg] or PBS performed in paraffin liver sections (*n* = 6–8/group). **(B)** Liver-to-body weight (LW/BW) ratio in female and male mice after acute alcohol injury (*n* = 5–8/group). Serum **(C)** ALT and **(D)** ALP levels in female and male mice (*n* = 7–8/group). **(E)** TUNEL staining was performed in liver cryosections of female and male mice. **(F)** Quantification of TUNEL positive cells was done and graphed (*n* = 3/group;*p < 0.05, ***p < 0.001, ^#^ *p* < 0.05).

The disruption of the intestinal barrier during alcohol exposure is related to liver injury (Sambrotta et al., 2014; Roychowdhury et al., 2019). Since experimental binge EtOH exposure impaired intestinal barrier integrity, we subsequently investigated the effects of acute alcohol injury on the liver. Light microscopy revealed normal lobular architecture with sinusoidal hepatic cords and typical liver architecture in PBS-treated mice (Figure 2A). In contrast, the structure of the hepatic lobules was disordered in mice with acute alcohol injury, with noticeable swelling around the central vein. The cytoplasm was translucent, exhibiting ballooning degeneration, some extent of hepatocellular necrosis, and visible inflammatory cell infiltration (CD11b^+^ cells). Interestingly, female livers exhibited extensive degeneration of hepatocytes (eosinophil cytoplasm) and nuclear lysis (Figure 2A). In line with these data, the LW/BW ratio of EtOH-treated female mice was significantly higher compared with PBS-treated mice (Figure 2B; Supplementary Figure S2A,B).

Next, serum markers of liver damage were evaluated. ALT levels were increased in male EtOH-treated mice compared with female or vehicle-treated mice (Figure 2C). ALP–a marker of cholestasis–was elevated in male mice after acute alcohol injury, while a tendency toward increased ALP was observed in female mice compared with PBS-treated animals (Figure 2D). Alcohol triggers hepatocyte cell death; thus, we investigated cell death in the liver using TUNEL. The percentage of TUNEL-positive cells increased after acute alcohol injury in female livers and, to a lesser extent, in male mice. In contrast, no relevant cell death was found in PBS-treated mice (Figures 2E,F).

**FIGURE 3:**
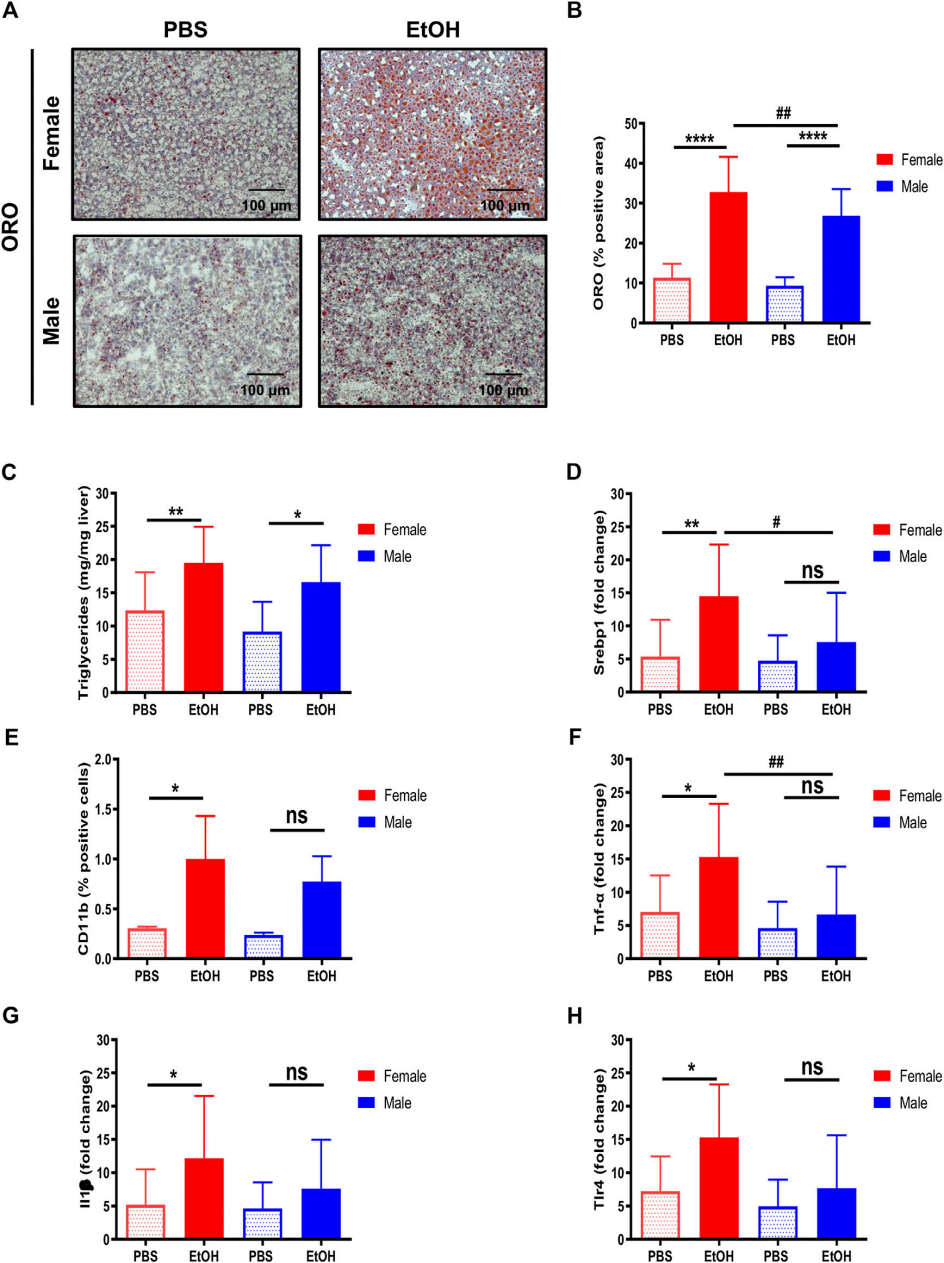
Hepatic lipid accumulation and inflammation are characteristic of female mice. **(A)** ORO staining performed in liver cryosection of female and male C57BL/6J mice treated with EtOH [6 g/kg] or PBS. **(B)** Quantification of lipid droplets by ORO positive area was done and graphed (*n* = 5–6/group). **(C)** Quantification of hepatic triglycerides was done and graphed (*n* = 8/group). **(D)** *Srbp-1* mRNA expression was determined by qPCR and normalized to the amount of GAPDH in the liver of female and male mice (*n* = 5–8/group). **(E)** Quantification of CD11b positive cells was done and graphed (*n* = 3/group). **(F)** *Tnf-α*, **(G)** *Il1β*, and **(H)** *Tlr4* mRNA expression determined by qPCR and normalized to the amount of *gapdh* in liver of female and male mice (*n* = 4–10/group; *p < 0.05, ****p < 0.0001, ^#^ *p* < 0.05, ^##^ *p* < 0.01).

Ethanol is metabolized in the liver by hepatocytes, being a primary inducer of liver injury and hepatic steatosis (Diehl et al., 1988; Ji et al., 2006; Kwon et al., 2014; Steiner and Lang, 2017). The staining of neutral lipids with ORO staining indicated an increase of lipid accumulation in livers of female and, to a lesser extent, of male mice, challenged with experimental acute alcohol injury (Figures 3A,B). Moreover, quantification of hepatic triglycerides showed an increase in both female and male animals, and activation of srebp-1, associated with increased expression of lipogenic genes, was also induced by EtOH predominantly in female mice (Figures 3C,D).

Since immune infiltration and inflammation are characteristic of alcohol-induced liver injury, quantification of CD11b-positive cells and markers of inflammation, including *tnf-α*, *il1β*, and *tlr4*, were also evaluated. The number of CD11b positive cells was increased in female livers (Figure 3E), in agreement with the upregulation of mRNA transcripts for *tnf-α*, *il1β*, and *tlr4* observed in female mice (Figures 3F–H). In summary, our data suggest that female mice are more sensitive to liver injury due to acute alcohol injury in terms of steatosis and inflammation.

### Assessment of Acute Alcohol Injury-Derived Damage in an *In Vitro* Model

In order to reproduce an *in vitro* model of the intestinal epithelial barrier, Caco-2 cells were plated and regularly monitored visually using a light microscope. First, Caco-2 cells were challenged with EtOH [0–100 mM], which caused cytoplasmic retractions, cell shrinkage, and cell death. High EtOH concentrations evidenced loss of cell-cell contact and detachment after 24 h EtOH exposure as observed under the visible light (Supplementary Figure S3A,B). Cell viability tested by CCK8 showed decreased cell viability along with increasing concentrations of EtOH (Supplementary Figure S3C). These data were corroborated using TUNEL staining, which also showed an elevation of the number of Caco-2 TUNEL-positive cells with increasing concentrations of alcohol (Supplementary Figure S3B,D).

As an *in vitro* model of hepatocytes, we also used the well-characterized HepaRG hepatocyte human cell line to understand the effect of alcohol exposure in the liver. HepaRG cells were challenged by exposure to EtOH [0–100]. The loss of cell architecture was evident with a concentration of 50 mM EtOH (Supplementary Figure S3A). Cell viability assay revealed a decrease of alive cells proportionally associated with increasing concentrations of EtOH (Supplementary Figure S3B,C). Moreover, TUNEL staining also confirmed that the number of dead HepaRG cells increased after exposure to EtOH (Supplementary Figures S3D). Besides, lipid deposition evaluated by ORO staining showed higher lipid accumulation with increasing EtOH concentrations (Supplementary Figures S3C,F).

### Intestinal Epithelial Cells Secrete Factors That Modulate Hepatocyte Injury During Acute Alcohol Injury

**FIGURE 4:**
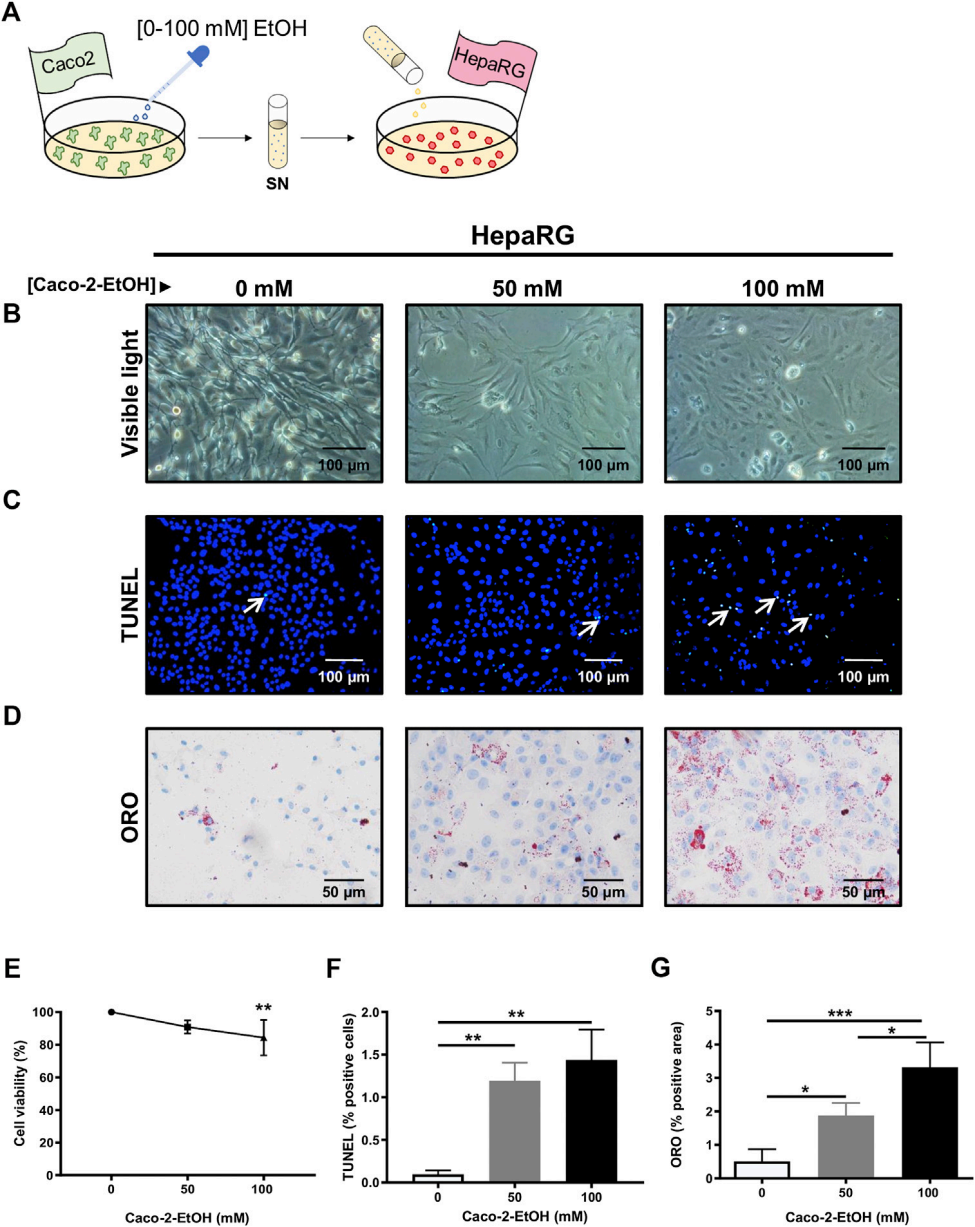
HepaRG cells challenged with supernatant from EtOH-treated Caco-2 cells displayed exacerbated cell damage. **(A)** Experimental *in vitro* model of the effect of acute alcohol injury on intestinal epithelial permeability and on liver injury: cultures of HepaRG cells were exposed to the supernatant from Caco-2 cells that had been previously treated with different concentrations of EtOH [0–100 mM]. **(B)** HepaRG cells exposed to the supernatant of Caco-2 cells treated with EtOH [0–100 mM] showed by visible light (*n* = 4/group). **(C)** TUNEL staining was performed in HepaRG cells exposed to the supernatant of Caco-2 cells treated with EtOH [0–100 mM]. **(D)** Oil Red O (ORO) staining was performed in HepaRG cells exposed to the supernatant of Caco-2 cells treated with EtOH [0–100 mM]. **(E)** Cell viability of HepaRG cells challenged with supernatant from EtOH-treated Caco-2 cells determined by CCK8 (*n* = 6/group). **(F)** Quantification of TUNEL positive cells HepaRG cells exposed to supernatant from EtOH-treated Caco-2 cells (*n* = 3/group). **(G)** Quantification of lipid droplets by ORO positive area in HepaRG cells exposed to supernatant from EtOH-treated Caco-2 cells (%) (*n* = 3–4/group; *p < 0.05, ***p < 0.001).

To replicate the effect of acute ethanol-derived injury on intestinal epithelial permeability and its impact on liver cell injury, 85% confluent HepaRG cells cultured in 12-well plates were challenged with 1 ml of supernatant from Caco-2 cells treated with EtOH [0–100 mM] (Figure 4A). The viability of HepaRG cells decreased at the higher concentrations of EtOH in the Caco-2 supernatant (Figures 4B,E), and these data correlated with the percentage of TUNEL positive cells (Figures 4C,F). Increased lipid accumulation, as assessed by ORO staining, was also found in HepaRG cells challenged with the supernatant of EtOH-treated Caco-2 cells (Figures 4D,G). These data indicate that the release of mediators from (IECs) might be responsible for hepatocyte injury during acute alcohol injury.

### Extracellular Vesicles Released From Intestinal Epithelial Cells Trigger Hepatocyte Damage *In Vitro*

It has been well-documented that the release of EVs, produced under pathological conditions, might be responsible for cell damage. To understand whether the release of EVs by IECs might trigger liver damage during acute alcohol injury, we tested this hypothesis by isolating EVs from our *in vitro* model of the intestinal epithelial barrier.

**FIGURE 5:**
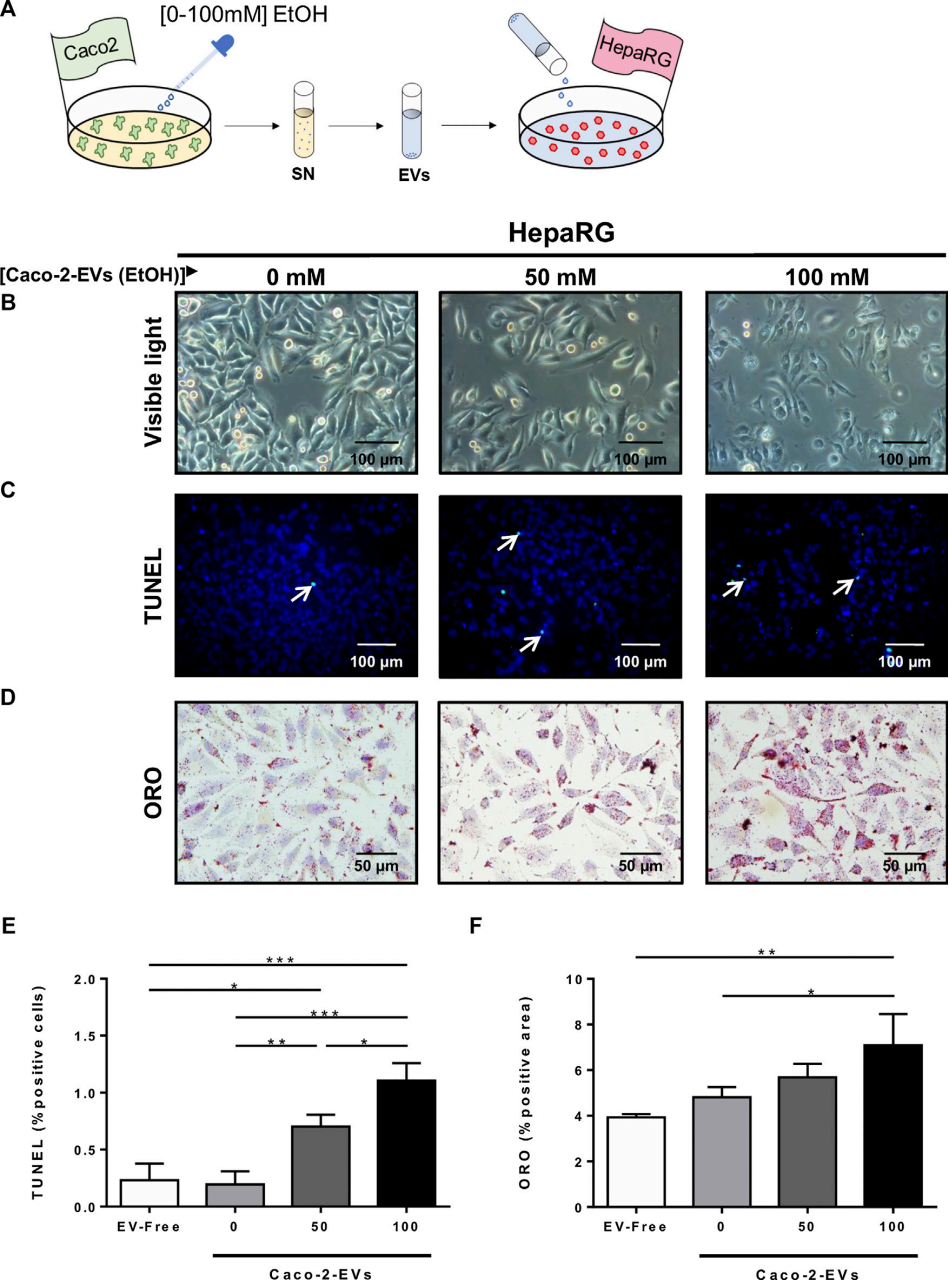
Extracellular vesicles released from IECs trigger hepatocyte cell damage *in vitro*. **(A)** Cultures of HepaRG cells were treated with EVs isolated by ultracentrifugation from the supernatant of Caco-2 cells previously exposed to different concentrations of EtOH [0–100 mM]. **(B)** Morphology analysis of HepaRG cells by visible light. **(C)** TUNEL staining was performed in HepG2 cells exposed to EVs from EtOH-pretreated Caco-2-cells. **(D)** ORO staining performed in HepaRG cells challenged with EVs from EtOH-pretreated Caco-2-cells. **(E)** Quantification of TUNEL positive HepaRG cells after challenge with EVs isolated from the supernatant of Caco-2 cells previously treated with different concentrations of EtOH [0–100 mM] (*n* = 3/group). **(F)** Quantification of lipid droplets by ORO positive area in HepaRG cells challenged with EVs isolated from the supernatant of Caco-2 cells previously treated with different concentrations of EtOH [0–100 mM] (*n* = 3/group; *p < 0.05, ***p < 0.001).

Cell cultures of differentiated hepatocytes were exposed to EVs treated with EtOH [0–100 mM] (Figure 5A). HepaRG cells changed the morphology and lost the polarity when challenged with EVs from EtOH-pretreated Caco-2 cells (Figure 5B).

Concomitant with these changes, the cell viability of HepaRG cells decreased, as observed by the increase of positive TUNEL cells after *in vitro* acute alcohol injury (Figures 5C,E). Next, we measured lipid deposition in hepatocytes in response to EVs released by EtOH-pretreated Caco-2 cells. Interestingly, EVs from EtOH-pretreated Caco-2 cells caused an increase in lipid deposition of differentiated hepatocytes (Figures 5D,F), suggesting a possible role for EVs released by IECs in liver damage during acute alcohol injury.

### Characterization of Extracellular Vesicles During Acute Alcohol Injury *In Vitro* and *In Vivo*

**FIGURE 6:**
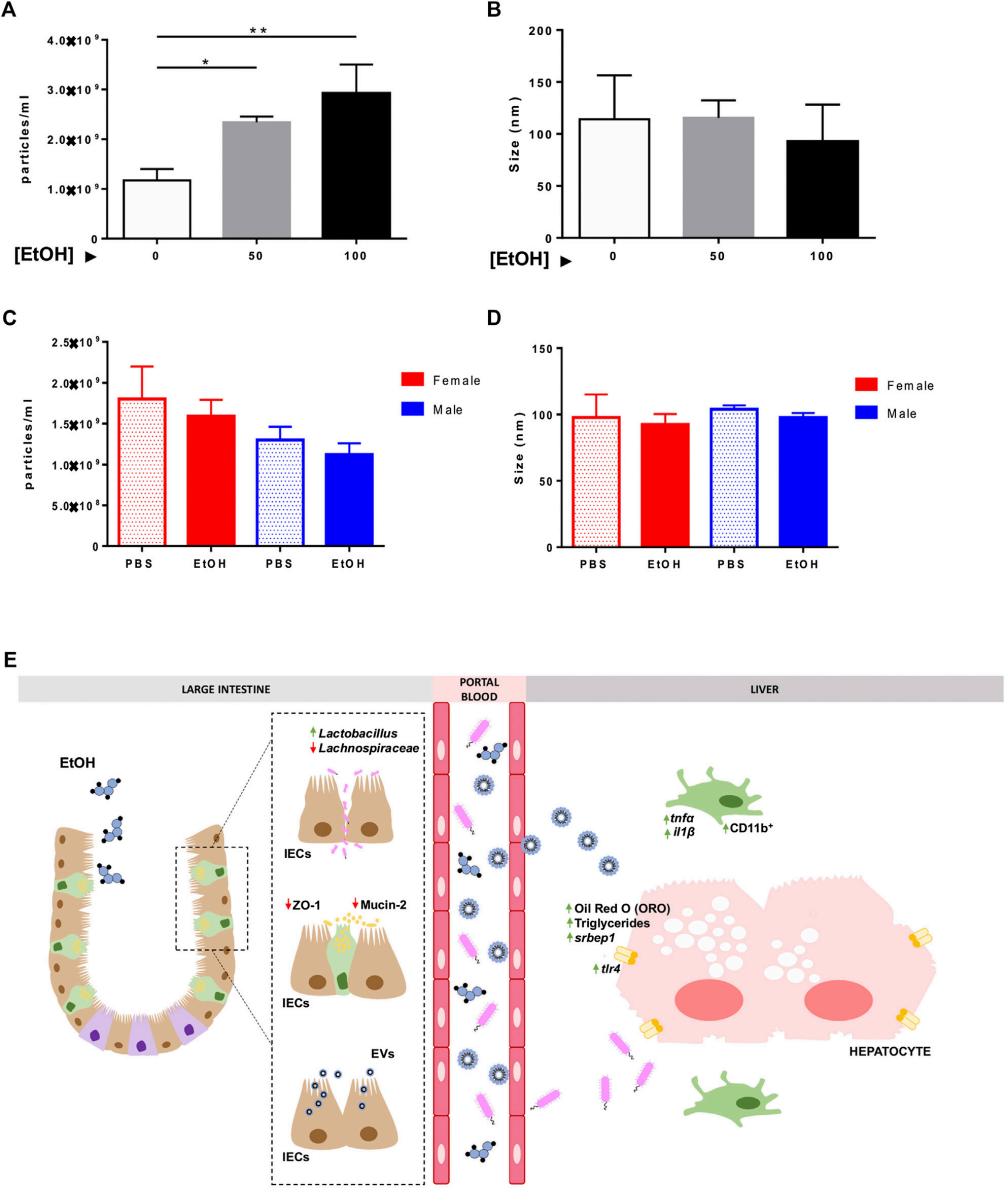
Characterization of extracellular vesicles *in vitro* and *in vivo*. Isolated EVs from the supernatant of Caco-2 cells challenged with different concentrations of EtOH [0–100 mM] were measured by NTA, and **(A)** particles/ml and **(B)** size of EVs were represented. Isolated EVs were extracted from the serum of female and male mice treated with EtOH or PBS, and they were analyzed using NTA. **(C)** Particles/ml and **(D)** size of EVs were graphed (*n* = 3/group; *p < 0.05, **p < 0.01). **(E)** Schematic representation of the pathophysiological events that occur during acute alcohol exposure. Acute alcohol ingestion triggers damage to the intestinal epithelial barrier. Increased FITC-Dextran, disruption in tight junctions (ZO-1), and loss of mucosa (MUCIN-2) and alteration of the gut microbiota (increased *Lactobacillus* and decreased Lachnospiraceae) indicate damage to the intestinal epithelial barrier and gut dysbiosis. Translocation of microbial bacterial products through the leaky gut causes overexpression of *tlr4* and intestinal-derived inflammation in the liver–presence of CD11b-positive cells, *tnf-α*, and *il1β*, and mild steatosis and lipid accumulation. Our *in vitro* experiments suggest that ethanol triggers the release of EVs by intestinal epithelial cells (IECs), which exert a deleterious effect on hepatocyte viability and lipid accumulation.

EVs were isolated from the supernatant of Caco-2 cells exposed to EtOH [0–100 mM] and from serum extracted from the portal vein of mice challenged to experimental acute alcohol injury, respectively, using a sequential ultracentrifugation method. Dynamic Light Scattering analysis showed the size distribution by intensity, confirming the existence of different populations of EVs (Supplementary Table S2), and NTA validated the morphology, size, and concentration of EVs in the diverse experimental groups (Figure 6 and Supplementary Figures S5B–D). Although the concentration of particles was significantly increased to 2.34 E^9^ ± 1.14 E^8^ and 2.9 E^9^ ± 5.82 E^8^ particles/ml in the supernatant of Caco-2 cells exposed to 50 and 100 mM EtOH, respectively, compared with the control group 1.17 E^9^ ± 2.31 E^8^ (Figure 6A), no differences were found in the size of EVs upon EtOH exposure (Figure 6B).

Next, we measured the number of particles in serum extracted from mice subjected to experimental acute alcohol injury. While no differences were found in the number of particles/ml between PBS and EtOH-treated animals, the concentration of particles was 1.12 E^9^ ± 1.40 E^8^ and 1.59 E^9^ ± 2.00 E^8^ in EtOH-fed male and female mice, respectively, compared with PBS-injected male and female mice 1.30 E^9^ ± 1.61 E^8^ and 1.80 E^9^ ± 3.99 E^8^. These results might indicate a tendency toward a reduced particle concentration in male animals after acute alcohol exposure (Figure 6C). NTA showed mean particle size of 90 nm in serum samples consistent with the size of exosomes (Figure 6D).

## Discussion

Scientific research on alcohol abuse has traditionally focused on the mechanisms of chronic toxicity, given its financial burden and societal costs. More recently, acute alcohol injury has emerged as a social problem since binge drinking is alarmingly increasing both in women and in men (Dolganiuc and Szabo, 2009; Mcketta and Keyes, 2019). However, the mechanisms by which acute alcohol injury affects human health are not fully understood. Thus, there is a need for preclinical and translational studies focused on the effects of binge alcohol drinking.

In the present study, we first used a suitable *in vivo* model for assessing the effects of a single binge episode by administering 6 g/kg b.w. of EtOH to mice, based on previous studies (Carson and Pruett, 1996).

Intestinal barrier function is key to preventing the alcohol-induced inflammation locally and systemically. Goblet cells in the intestinal epithelium produce protective trefoil factors and mucins, which are abundantly core glycosylated and either localized to the cell membrane or secreted into the lumen to form the mucous layer (Shao et al., 2018). Decreased expression of MUCIN-2 protein in the large intestine of female and male mice was observed after acute alcohol exposure. Interestingly, *mucin-2* knockout mice are protected against alcohol feeding-induced dysbiosis (Hartmann et al., 2013), likely due to higher expression of antimicrobial peptides.

Additionally, paracellular permeability in the intestinal epithelium sealed by TJs (Shao et al., 2018) was disrupted in the colon of EtOH-treated female and male mice, in which the loss of ZO-1 was characteristic. These results are in agreement with previous observations reporting that chronic-binge ethanol feeding impaired intestinal TJs (Cresci et al., 2017). Moreover, occludin deficiency increases susceptibility to EOH-induced mucosal dysfunction and liver damage in mice (Mir et al., 2016).

Alcohol ingestion causes intestinal bacterial overgrowth and changes in microbial composition in preclinical models as well as in patients with alcohol use disorder (Sarin et al., 2019). In our study, one alcohol binge increased the *Lactobacillus* phylum and decreased the Lachnospiraceae family. A decrease in *Lactobacillus* species and Lachnospiraceae family has been reported in alcohol consumption/feeding and alcoholic cirrhosis (Bajaj et al., 2012; Sarin et al., 2019). However, *Lactobacillus* species are increased in hepatic steatosis (Jang et al., 2019), which was observed in acute alcohol injury-exposed animals, and bacterial microbiota also change with the progression of alcoholic liver disease. Moreover, in a similar preclinical model that used a lower EtOH dosage (3 g/kg b.w.), Chen and colleagues (2016) reported no changes in intestinal microbiota and relative abundance of *Lactobacillus* and Firmicutes. Moreover, it is very likely that *Lactobacillus* elevation acts to counteract lipid metabolism imbalance as previously reported (Martin et al., 2008). Overall, these data suggest that alterations to the gut mucus layer, together with intestinal hyperpermeability and bacterial overgrowth, trigger bacterial translocation. Therefore, microbial products that reach the liver might contribute to hepatic injury during acute alcohol exposure.

Hepatic steatosis develops acutely in most individuals that consume even moderate amounts of alcohol. Steatosis changes are also seen in rodent models of binge drinking (Massey and Arteel, 2012). Although steatosis is an inert pathology *per se*, it sensitizes the liver to injury caused by a second insult. Binge drinking is a major risk factor for advanced liver disease (Ventura-Cots et al., 2017). Ethanol-treated mice displayed increased hepatic lipid accumulation, and this effect was more noticeable in female animals. This phenomenon was associated with increased immune infiltration (CD11b putative macrophages) and pro-inflammatory mediators of hepatic inflammation (e.g., *tnf-α*, *il1β*), which contribute to alcohol-related liver injury (Kawaratani et al., 2013; Bala et al., 2014; Rocco et al., 2014; Cresci et al., 2017).

Ethanol pre-exposure can prime Kupffer cells to lipopolysaccharides stimulation, resulting in enhanced *tnf-α* release, and binge drinking can impair the immune response via alteration of *tlr4* signaling (Massey and Arteel, 2012; Bala et al., 2014), as observed in our acute alcohol injury model. Moreover, our data demonstrated a significant increase in cell death in EtOH-treated mice, corroborated by elevated serum markers of liver injury (e.g., ALT). In agreement with other publications (Wagnerberger et al., 2013; Cresci et al., 2017), the more pronounced liver damage observed after acute alcohol injury in the liver of female mice may result from increased activation of TLR4-dependent signaling in this gender. However, in-depth studies need to be performed to shed light on the activation of specific molecular mechanisms that might be sex-dependent in the gut-liver axis.

Gender-related differences in total liver (ADH) and aldehyde dehydrogenase activity among different animal species have been observed in many studies. The differential liver injury between female and male animals could be explained by various factors, including the lower activity of class I and II ADH isoenzymes in females (Chrostek et al., 2003). This difference could help explain the fact that after men and women ingest the same dose of alcohol, women have higher BAC levels and increased injury. Corrao and colleagues (1998) reported that the same amount of average alcohol consumption was related to a higher risk of liver cirrhosis in women than in men. In contrast, women displayed slower gastric metabolism. Additionally, the levels of hormones, such as estrogen, might also influence several of the above factors (Wagnerberger et al., 2013).

Next, we sought to mechanistically approach the pathomechanisms underlying acute alcohol injury in the context of the gut-liver axis by modeling our experimental model *in vitro*. First, the effects of EtOH were assessed in Caco-2 cells, a human cell line of IECs. Concomitant with Wang’s study (Wang et al., 2014), acute alcohol injury in culture decreased cell viability and exacerbated cell death, even though our results were collected 24 h after challenge (Dolganiuc and Szabo, 2009). Furthermore, EtOH caused changes in cell death and lipid deposition in HepaRGs, a human hepatoma cell line, in a dose-dependent manner, data that agree with previous publications (Tuoi Do et al., 2011). However, these authors observed that exposure to 100 mM ethanol significantly raised caspase 3/7 activity between 48 and 72 h, suggesting that apoptosis might occur later in time in culture. However, *in vivo*, we observed TUNEL-related cell death 8 h after acute ethanol injury. Others reported maximal apoptotic rate levels 4 h after ethanol exposure but TUNEL-positivity from 1 to 9 h (Yun et al., 2014), which might be increased levels of expression of CYP2E1 at these times after acute ethanol-derived tissue injury.

Accumulating evidence supports a role for EVs in regulating hepatic function. The gut-liver axis communication was modeled *in vitro* by challenging HepaRG cells with supernatant of Caco-2 cells. Human HepaRG hepatocytes treated with the supernatant of EtOH-pretreated Caco-2 cells dramatically changed their morphology, increased cell death, and accumulated lipids, as it occurs *in vivo*. This set of results suggested that the release of mediators by IECs may play a crucial role in the initiation and development of liver injury, and thus hepatocyte might uptake EVs as observed in models of viral hepatitis, partial hepatectomy and ischemia-reperfusion injury (Hirsova et al., 2016).

Recent studies pointed to a defined group of biological nanovesicles, namely exosomes or extracellular vesicles, as a key player in modulating the deleterious effects of alcohol in different tissues (Eguchi and Feldstein, 2018). Since our data indicated that EVs might be potential communicating tools in the gut-liver axis, we performed an *in vitro* model whereby EVs secreted by IECs (Caco-2 cells) in response to EtOH were added to HepaRG hepatocytes. EtOH significantly increased the number of nanoparticles released by Caco-2 cells, which were challenged to HepaRG cells. Decreased cell viability and high lipid accumulation were observed in this cell line of human hepatocytes, concomitant with our *in vivo* results.

In parallel, EVs isolated from portal blood of EtOH- and PBS-treated mice were collected and characterized. Our goal was to demonstrate that EVs released specifically by IECs can be used as potential biomarkers of acute alcohol injury. Our data showed a mean size of 90 nm in serum samples consistent with the size of EVs. However, hepatocytes also release EVs that are detected in blood as well, as previously reported (Eguchi et al., 2017), suggesting that the gut-liver axis is a bidirectional communication pathway.

Our results show that mice develop alterations in the gut-liver axis in response to experimental acute alcohol injury. Overall, alterations in the intestinal epithelial barrier associated with increased permeability, a thinner mucous protective layer, and changes in gut microbiota were evident. Interestingly, female mice were more prone to hepatic injury in response to a single binge episode, specifically in markers of steatosis and inflammation. Moreover, we showed that the release of mediators by IECs might have an impact on liver cells during acute alcohol injury, and demonstrated the presence and the effects of EVs both *in vivo* and *in vitro* (Figure 6E). These findings further deepen in the mechanisms triggered by acute alcohol exposure and open new therapeutic windows.

## Data Availability Statement

The datasets generated for this study can be found in ENA, Accession No. PRJEB40638.

## Ethics Statement

Animal studies were approved by the Consejería de Medio Ambiente, Administración Local y Ordenación del Territorio (PROEX-154/16).

## Author Contributions

AL-P and LM carried out the experiments and drafted the manuscript. JP provided funds for the study. NL-A, FH, and KZ performed experiments. SS, RV-V, and LB performed the microbiota studies. YN contributed to the intellectual work and provided experimental techniques. BM-A, LM-G, and AC contributed with the extracellular vesicles characterization. IA, MG, JV, RB, BM-A, and EM-N conducted experiments and/or provided pivotal intellectual and YN and FJ supervised, and analyzed the experiments, provided the funding and drafted the manuscript

## Funding

This work was supported by the MINECO Retos SAF2016-78711, SAF2017-87919-R, EXOHEP-CM S2017/BMD-3727, NanoLiver-CM Y2018/NMT-4949, ERAB Ref. EA 18/14, AMMF 2018/117, UCM-25-2019 and COST Action CA17112, the German Research Foundation (SFB/TRR57/P04, SFB 1382-403224013/A02, and DFG NE 2128/2-1). FC and YN are Ramón y Cajal Researchers RYC-2014-15242 and RYC-2015-17438. FC is a Gilead Liver Research 2018. KZ is a recipient of a Chinese Scholarship Council (CSC). BK20170127 from the Natural Science Foundation of Jiangsu Province to JP

## Conflict of Interest

The authors declare that the research was conducted in the absence of any commercial or financial relationships that could be construed as a potential conflict of interest.

## Abbreviations

ADH, alcohol dehydrogenase; ALDH, aldehyde dehydrogenase; BAC, blood alcohol concentration; DLS, dynamic light scattering; EtOH, ethanol; EVs, extracellular vesicles; H&E, hematoxylin and eosin; IF, immunofluorescence; IECs, intestinal epithelial cells; IL-1β, interleukin-1β; KCs, Kupffer cells; LPS, lipopolysaccharides; LW/BW, liver weight vs. body weight; NTA, nanoparticle tracking analysis; ORO, oil red O; qRT-PCR, quantitative real-time PCR; Srebp-1, sterol regulatory binding protein-1; Tnf-α, tumor necrosis factor-alpha; TJs, tight junctions; Tlr-4, toll-like receptor-4; ZO-1, zone occludens-1.
