# Supplementary material for: Intestinal Epithelial Cell-Derived Extracellular Vesicles Modulate Hepatic Injury via the Gut-Liver Axis During Acute Alcohol Injury
Source: Front Pharmacol. 2020 Dec 21;11:603771. doi: 10.3389/fphar.2020.603771 (PMC7779758; doi:10.3389/fphar.2020.603771)
Supplement: Supplementary file 1 [file datasheet1.pdf]

SUPPLEMENTARY MATERIAL

Intestinal epithelial cells-derived extracellular vesicles (EVs) modulate hepatic injury via the gut-liver axis during acute alcohol injury

Arantza Lamas-Paz, Laura Morán, Jin Peng, Beatriz Salinas, Nuria López-Alcántara, Svenja Sydor, Ramiro Vilchez-Vargas, Iris Asensio, Fengjie Hao, Kang Zheng, Beatriz Martín-Adrados, Laura Moreno, Angel Cogolludo, Manuel Gómez del Moral, Lars Bechmann, Eduardo Martínez-Naves, Javier Vaquero, Rafael Bañares, Yulia A. Nevzorova, Francisco Javier Cubero

SUPPLEMENTARY METHODS

Microbiota evaluation

Briefly, The extraction was performed using the QIamp DNA isolation Kit following the manufacturer´s instructions (Qiagen, Hilden, Germany) but including a mechanical lysis step using dry bead tubes (Mobio Laboratories Inc, Carlsbad, CA, USA) and Fast Prep^{TM}-24 instrument (MP Biomedical, SOLON, OH, USA) at 6.0 m/s for 45 s (two times). Amplicon libraries for sequencing were generated as previously described (Rath et al., 2017), where the V1-V2 region of the 16S rRNA gene was amplify after 20 PCR cycles using the 27F and 338R primers (Rath et al., 2017) and sequenced on a MiSeq (2 x 250 bp, illumine, Hayward, California, USA). All the fastQ files were analyzed using dada2 package in R (Callahan et al., 2016) and a unique table containing all

samples with the sequence reads and abundances was generated. Sequences were taxonomically annotated using ribosomal data project based on the naïve Bayesian classification (Wang et al., 2007) with a pseudo-bootstrap threshold of 80%. Relevant phylotypes were taxonomically annotated until species using the tool Sequence match of ribosomal dataproject and the name of the species were given if the sequence was identical to the sequence deposited in the database. Relative of abundances, in percentage of phylotypes were used for downstream analyses. Principal coordinates analysis was performed using Past4.02. Significant differences between a priori defined groups were evaluated using Permutation multivariate analysis of variance (PERMANOVA with 9999 permutations) and groups were considered significant different if the p value was <0.05. PERMANOVA was performed also in Past4.02. Statistically significant abundances of single phylotypes were calculated with Mann-Whitney test using the package exactRankTests of R. Only if the phylotype was more than 2% of abundance, it was considered for Mann-Whitney test.

SUPPLEMENTARY TABLES

Supplementary Table 1. Sequences of primers used for quantitative real-time PCR (qRT-PCR).

Gene	FW (5' – 3')	RV (5' – 3')
il1β	TGTGTGACGTTCCCATT	CAGCACGAGGCTTTTTTGTTG
Srebp1	GAGGCCAAGCTTTGGACCTGG	CCTGCCTTCAGGCTTCTCAGG
Tlr4	GCCTTTCAGGGAATTAAGCTCC	AGATCAACCGATGGACGTGTAA
Tnf	CCTCTTCTCATTCCTGCTTGTGG	GAGAAGATGATCTGAGTGTGAGG

gapdh	CAAGGTCATCCATGACAACTTTG	GTCCACCACCCTGTTGCTGTAG

Supplementary Table 2. Data from Dynamic light scattering (DLS) characterization of EVs

		z-average (nm)[a]	PdI (nm)[b]	Size[c] (nm)±SD
**Female**	**PBS**	71,36	0,696	107,7±48,85
	**EtOH**	192,9	0,208	116,6±49,64
**Male**	**PBS**	92,13	0,252	126,3±51,93
	**EtOH**	141,3	0,11	177,4±67,65

[a]Intensity-weighed harmonic mean hydrodynamic diameter (z-average diameter)

[b]Polydispersity index

[c]Average size of vesicle (particle)

SUPPLEMENTARY FIGURE LEGENDS

Supplementary Figure 1. Gut microbiota composition in experimental acute alcohol intoxication. (**A**) PCA analysis graph based on Bray-Curtis dissimilarity shows female and male mice treated with either PBS (Control) or EtOH (Ethanol). The analysis showed significantly increased Phyla 2 and 6 (P<0,005677) corresponding to *Lactobacillus* and significantly decreased *Lachnospiraceae* (Phyla 63; P<0,02941). (**B**) Relative abundance of the phylotypes shown in the PCA analysis.

Supplementary Figure 2. Changes in body weight and liver weight after AAI. (**A**) Body weight (BW) (g). (n=6-8). (**B**) Liver weight (LW) (g) (n=5-9). (#P < 0.05).

Supplementary Figure 3. Evaluation of acute alcohol cytotoxicity *in vitro*. (**A**) Caco-2 cells treated from 0 to 100 mM EtOH showed by visible light. (n=4). (**B**) TUNEL staining performed in Caco-2 cells challenged with EtOH [0-100 mM]. (**C**) Cell viability of Caco-2 cells determined by CCK8. (n=5-9). (**D**) Quantification of TUNEL positive cells in Caco-2 cells treated with EtOH [0-100 mM]. (n=3; *P < 0.05-****P<0.0001).

Supplementary Figure 4. Acute alcohol-induced cytotoxicity assessment *in vitro* on a human cell line that displays hepatocyte-like functions. (**A**) HepaRG cells challenged from 0 to 100 mM EtOH showed by visible light. (n=4). (**B**) TUNEL staining performed in HepaRG cells challenged with EtOH [0-100 mM]. (**C**) ORO staining performed in HepaRG cells challenged with EtOH [0-100 mM]. (**D**) Cell viability of HepaRG cells determined by CCK8. (n=6). (**E**) Quantification of TUNEL positive cells HepaRGs treated with EtOH [0-100 mM]. (n=2-3). (**F**) Quantification of lipid droplets by ORO positive area in HepaRG cells treated with EtOH [0-100 mM]. (n=2-3; **P < 0.01- ***P < 0.001).

Supplementary Figure 5. Isolation and characterization of intestinal epithelial cells-derived EVs in response to acute alcohol intoxication *in vitro*. (**A**) Cells were grown in EV-free medium as controls and visible light, TUNEL and ORO micrographs were taken. (**B-D**) Representative graph of nanoparticle tracking analysis (NTA) analysis of Trabecular Meshwork (TM) of EVs extracted from serum from female mice treated with PBS (**B**) and EtOH (**C**)

and male mice treated with PBS (**D**) and EtOH (**E**). In blue the averaged size of particle/concentration is annotated.

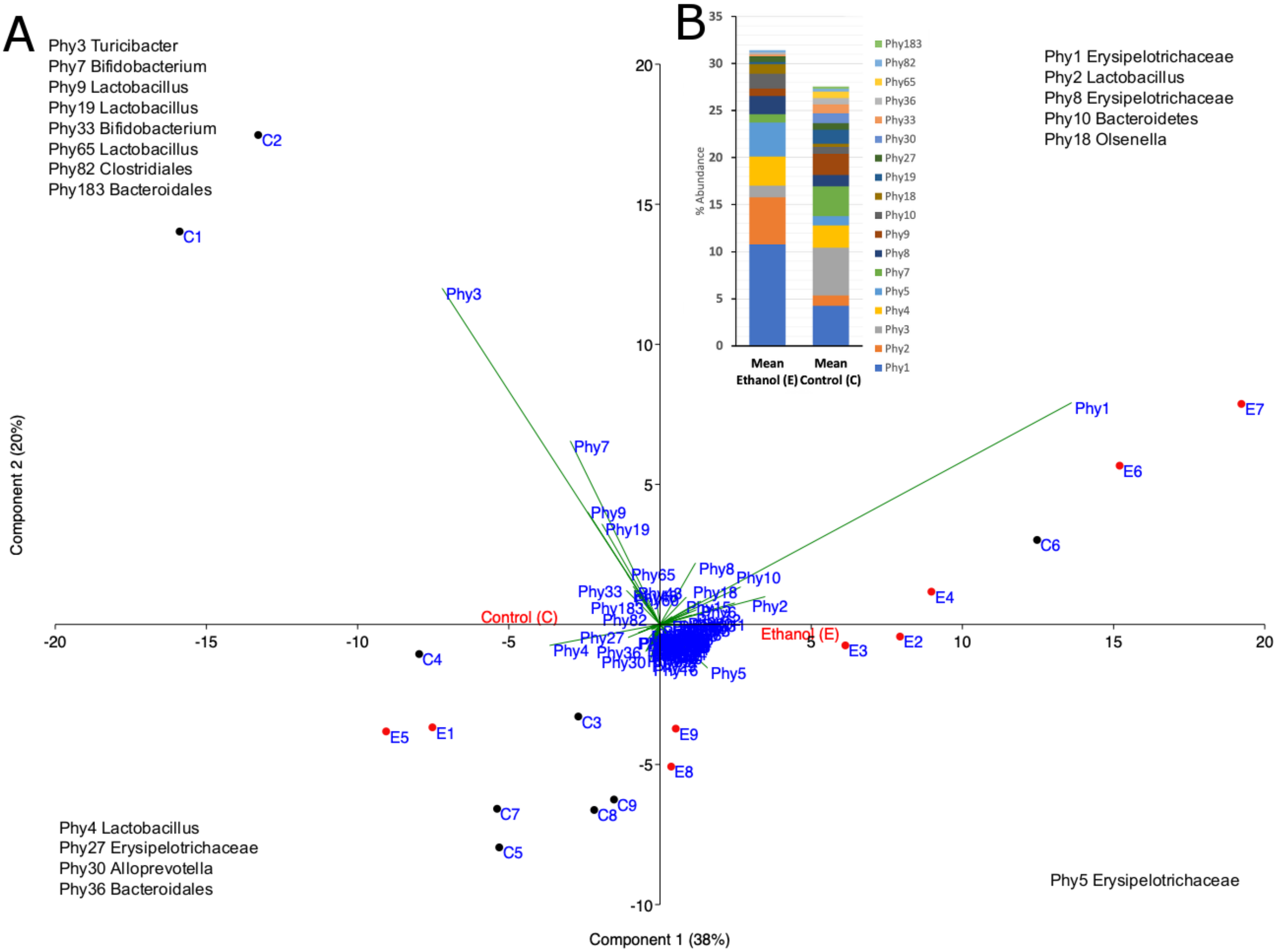
A
Phy3 Turicibacter
Phy7 Bifidobacterium
Phy9 Lactobacillus
Phy19 Lactobacillus
Phy33 Bifidobacterium
Phy65 Lactobacillus
Phy82 Clostridiales
Phy183 Bacteroidales
Phy1 Erysipelotrichaceae
Phy2 Lactobacillus
Phy8 Erysipelotrichaceae
Phy10 Bacteroidetes
Phy18 Olsenella
Phy4 Lactobacillus
Phy27 Erysipelotrichaceae
Phy30 Alloprevotella
Phy36 Bacteroidales
Phy5 Erysipelotrichaceae
Component 2 (20%)
Component 1 (38%)
Control (C)
Ethanol (E)
B
% Abundance
Mean Ethanol (E)
Mean Control (C)

Suppl. Fig. 1

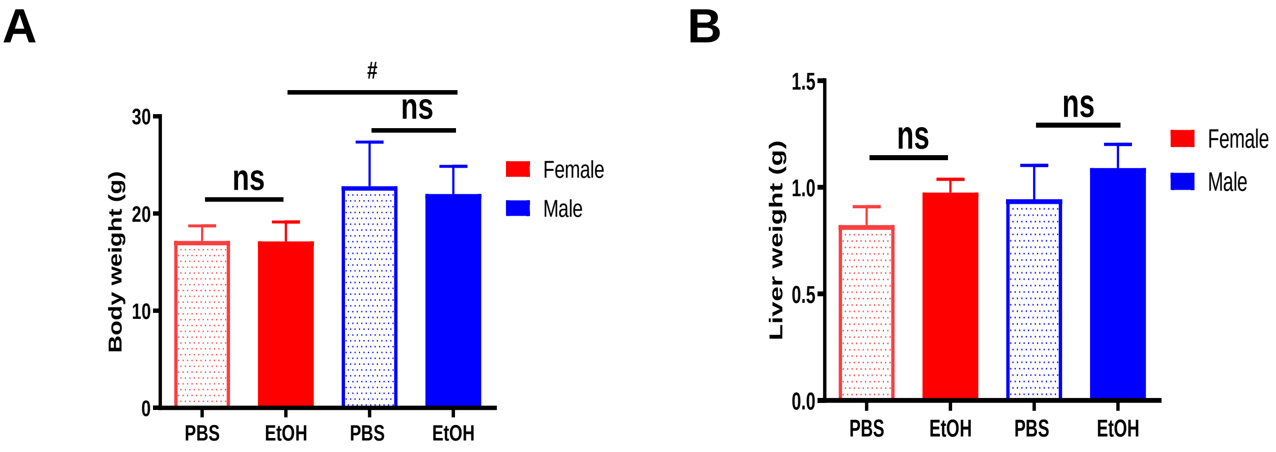

A
#
ns
ns
30
20
10
0
Body weight (g)
PBS
EtOH
PBS
EtOH
Female
Male
B
1.5
1.0
0.5
0.0
Liver weight (g)
ns
ns
PBS
EtOH
PBS
EtOH
Female
Male

Suppl. Fig. 2

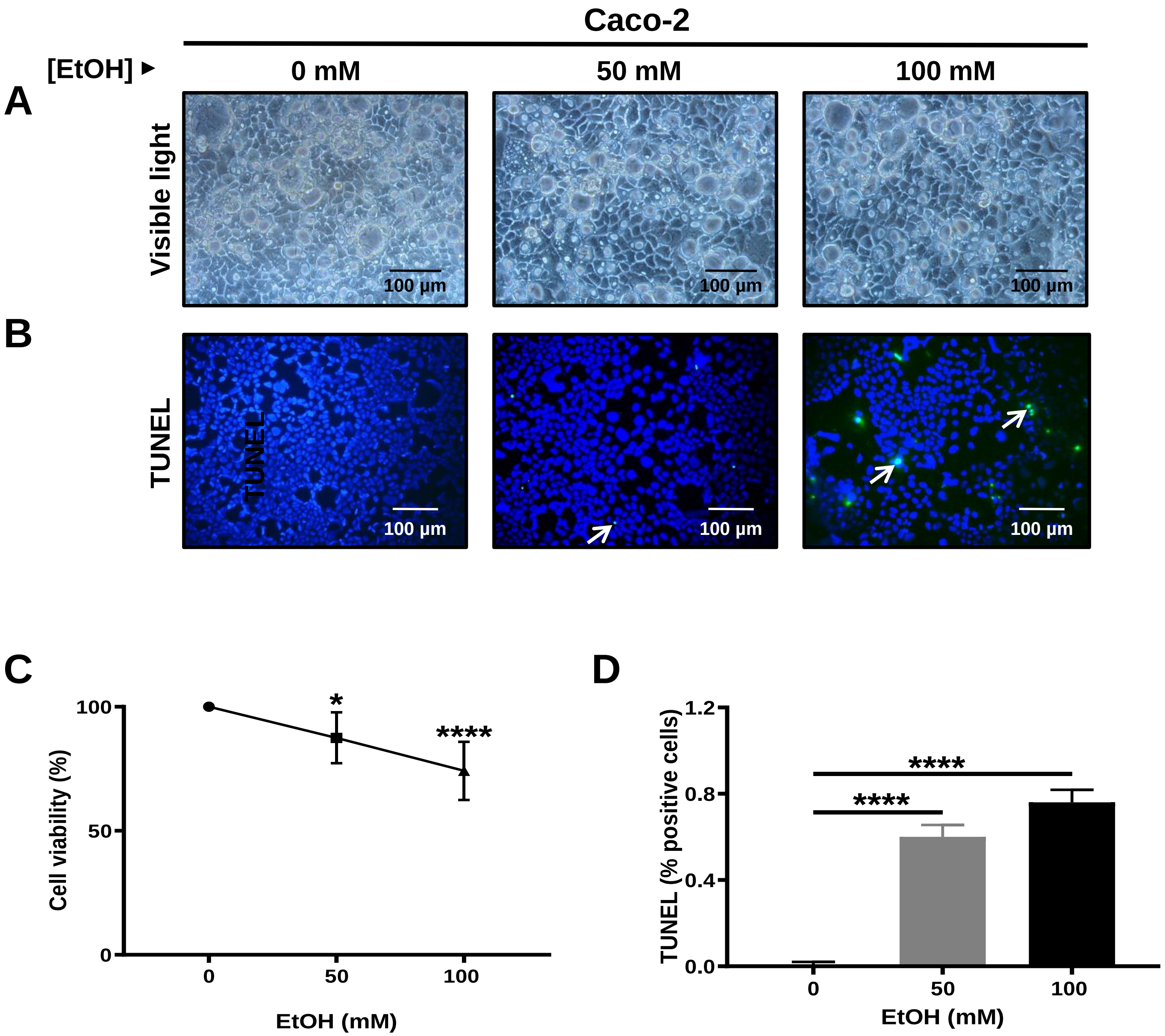

Caco-2
[EtOH] ▸
0 mM
50 mM
100 mM
A
Visible light
100 µm
100 µm
100 µm
B
TUNEL
100 µm
100 µm
100 µm
C
Cell viability (%)
100
50
0
0
50
100
EtOH (mM)
*
****
D
TUNEL (% positive cells)
1.2
0.8
0.4
0.0
0
50
100
EtOH (mM)
****
****

Suppl. Fig. 3

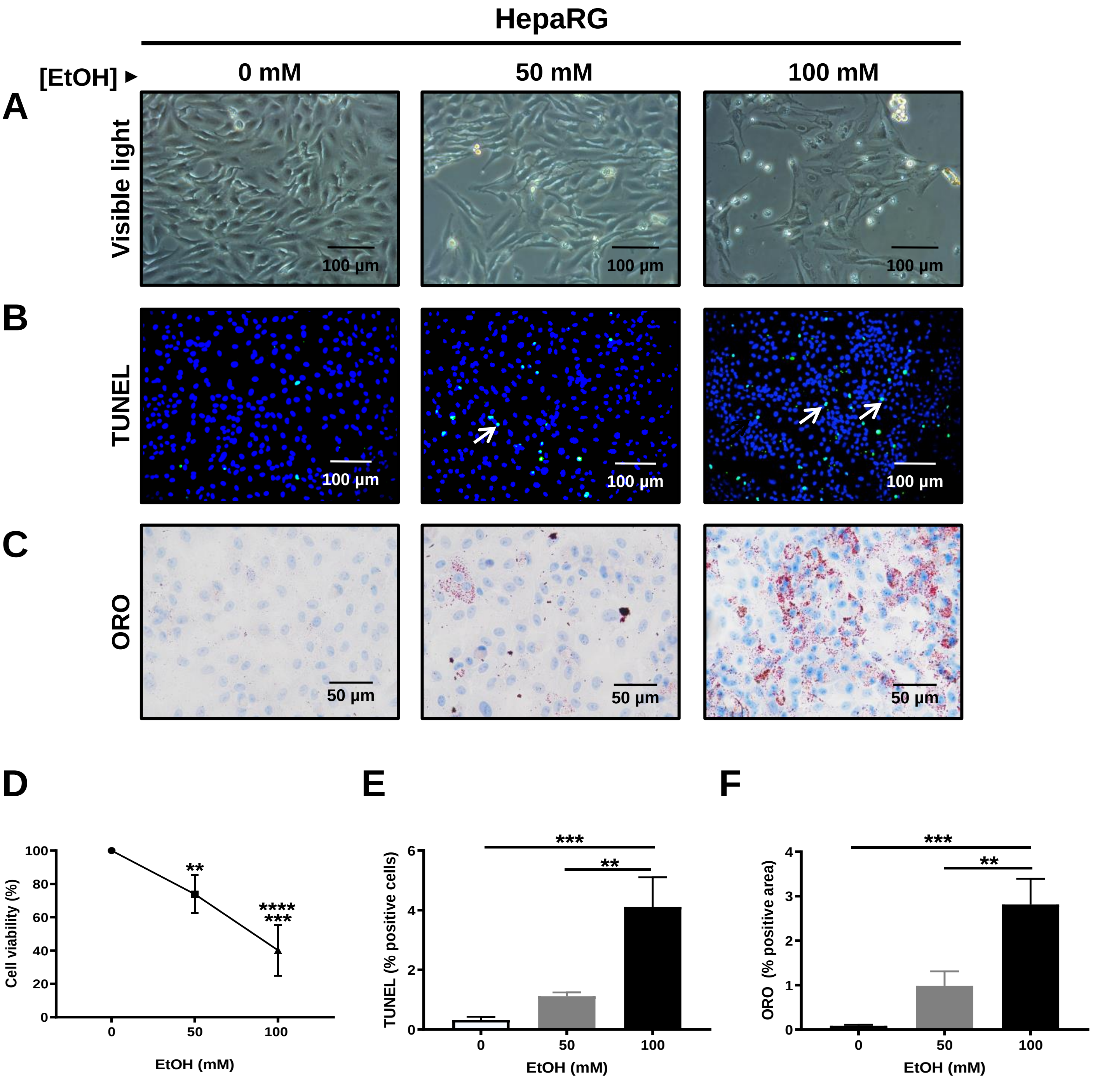

HepaRG
[EtOH] ▸
0 mM
50 mM
100 mM
A
Visible light
100 µm
B
TUNEL
C
ORO
50 µm
D
Cell viability (%)
100
80
60
40
20
0
**
****
***
EtOH (mM)
E
TUNEL (% positive cells)
F
ORO (% positive area)

Suppl. Fig. 4

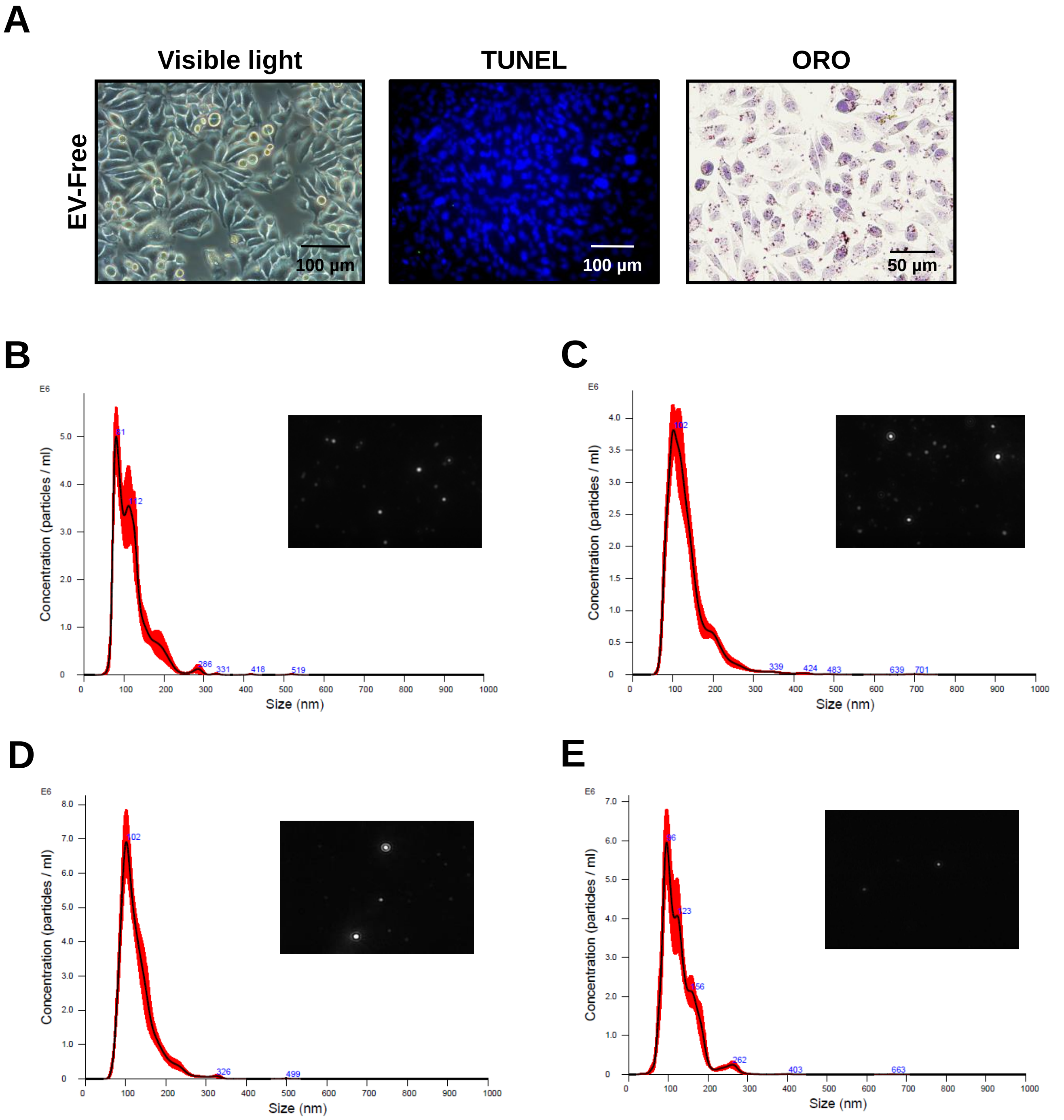
A
Visible light
TUNEL
ORO
EV-Free
100 µm
100 µm
50 µm
B
C
D
E
E6
Concentration (particles / ml)
Size (nm)
81
112
286
331
418
519
102
339
424
483
639
701
102
326
499
96
123
156
262
403
663

Suppl. Fig. 5
